# E-Cadherin Orthologues as Substrates for the Serine Protease High Temperature Requirement A (HtrA)

**DOI:** 10.3390/biom12030356

**Published:** 2022-02-24

**Authors:** Sabine Bernegger, Evelyn Hutterer, Urszula Zarzecka, Thomas P. Schmidt, Markus Huemer, Isabella Widlroither, Gernot Posselt, Joanna Skorko-Glonek, Silja Wessler

**Affiliations:** 1 Department of Biosciences and Medical Biology, Division of Microbial Infection and Cancer, Paris-Lodron University of Salzburg, 5020 Salzburg, Austria; sabine.bernegger@plus.ac.at (S.B.); evelyn.hutterer@plus.ac.at (E.H.); thomas.phi.schmidt@gmail.com (T.P.S.); markus.huemer1991@me.com (M.H.); isabella.widlroither@plus.ac.at (I.W.); gernot.posselt@plus.ac.at (G.P.); 2 Department of General and Medical Biochemistry, Faculty of Biology, University of Gdańsk, 80-308 Gdańsk, Poland; urszula.zarzecka@ug.edu.pl (U.Z.); joanna.skorko-glonek@biol.ug.edu.pl (J.S.-G.); 3 Cancer Cluster Salzburg and Allergy Cancer BioNano Research Centre, University of Salzburg, Hellbrunner Strasse 34, 5020 Salzburg, Austria

**Keywords:** HtrA, E-cadherin, infection, pathogens

## Abstract

*Helicobacter pylori* (*H. pylori*) expresses the serine protease and chaperone High temperature requirement A (HtrA) that is involved in periplasmic unfolded protein stress response. Additionally, *H. pylori*-secreted HtrA directly cleaves the human cell adhesion molecule E-cadherin leading to a local disruption of intercellular adhesions during pathogenesis. HtrA-mediated E-cadherin cleavage has been observed in response to a broad range of pathogens, implying that it is a prevalent mechanism in humans. However, less is known whether E-cadherin orthologues serve as substrates for bacterial HtrA. Here, we compared HtrA-mediated cleavage of human E-cadherin with murine, canine, and simian E-cadherin in vitro and during bacterial infection. We found that HtrA targeted mouse and dog E-cadherin equally well, whereas macaque E-cadherin was less fragmented in vitro. We stably re-expressed orthologous E-cadherin (Cdh1) in a CRISPR/Cas9-mediated *cdh1* knockout cell line to investigate E-cadherin shedding upon infection using *H. pylori* wildtype, an isogenic *htrA* deletion mutant, or complemented mutants as bacterial paradigms. In Western blot analyses and super-resolution microscopy, we demonstrated that *H. pylori* efficiently cleaved E-cadherin orthologues in an HtrA-dependent manner. These data extend previous knowledge to HtrA-mediated E-cadherin release in mammals, which may shed new light on bacterial infections in non-human organisms.

## 1. Introduction

Cadherins are key players in cell adhesion, regulation of tissue organization and morphogenesis [1]. In epithelial cells, E-cadherin represents a crucially important molecule in the establishment of intercellular adhesions and functions as a tumor suppressor. The domain structure of E-cadherin is characterized by an extracellular domain (EC), a transmembrane (TM) domain, and an intracellular domain (IC). The extracellular domain contains five tandemly repeated sequences (EC1-5). Calcium ions bind to negatively charged motifs located between the EC repeats to provide the adhesive properties of E-cadherin. The calcium-bound ectodomains of E-cadherin interact in *trans* with E-cadherin from opposing cells and in *cis* on the same cell, respectively, leading to the formation of connective dimers and E-cadherin clustering [2,3]. The IC domain of E-cadherin recruits cytoplasmic signaling proteins, including β-catenin and p120-catenin, which control the strength and integrity of the adhesion complex and regulate downstream signaling transduction pathways implicated in the actin cytoskeleton reorganization and cancer-associated gene transcription [4,5].

E-cadherin-based cell adhesion is a dynamic process. Cytoplasmic signal transduction pathways can interfere with the integrity of cadherin complexes leading to disruption of the cell adhesion machinery [6,7]. Further, downregulation of E-cadherin expression via transcriptional repression, loss-of-function mutations, or extracellular shedding have been connected with alterations at adhesive interfaces implicated in a variety of cellular processes such as cell polarity, proliferation, survival, and invasive growth [8,9,10,11]. A number of cellular proteases, including activated A Disintegrin And Metalloproteinase domain-containing protein 10 (ADAM10) or the matrix metalloproteinases MMP-3 and MMP-7, have been described that cleave-off the EC domain of E-cadherin on the cell surface [10,11]. In addition to these host factors, bacteria-derived serine proteases were identified as potent E-cadherin proteases with crucial roles in the progression of infectious diseases. Almost all bacteria express at least one homologue of the evolutionary conserved serine protease and chaperone High temperature requirement A (HtrA) family [12]. In *Escherichia coli* (*E. coli*), the HtrA proteases DegP, DegQ, and DegS serve as structural paradigms for HtrA proteins expressed by Gram-negative bacteria [13]. DegP and DegQ consist of an N-terminal signal peptide, which is responsible for periplasmic localization followed by a protease domain harboring the catalytic triad composed of histidine, serine, and aspartate residues. In the C-terminal region, DegP and DegQ contain two PDZ (postsynaptic density protein [PSD95], *Drosophila* disc large tumor suppressor [Dlg1], and zonula occludens-1 protein [ZO-1]) domains mediating protein-protein interactions, substrate recognition and substrate binding [14,15]. The activity of HtrA proteins is dependent on the formation of multimeric structures consistent of trimers as building blocks. HtrA assembles into inactive hexameric oligomers that are converted into proteolytic active oligomers consisting of 12–24 HtrA monomers upon substrate binding [15,16].

HtrA proteins were discovered as determinants in virulence of bacterial pathogens. The human class-I carcinogen *Helicobacter pylori* (*H. pylori*) secretes HtrA into the environment and mediates E-cadherin ectodomain cleavage [17,18,19]. In addition to the HtrA substrate E-cadherin, *H. pylori* HtrA also targets the tight junction proteins claudin-8 and occludin [20] as well as the desmosomal cadherin desmoglein-2 [21], implying that the main function of extracellular HtrA is to open intercellular adhesions of polarized epithelial cells. Consequently, *H. pylori* can transmigrate across the epithelium and translocate the virulence factor cytotoxin-associated gene A (CagA) at the basolateral domain of polarized epithelial cells [20]. HtrA proteins are expressed by many organisms. The human pathogens *Campylobacter jejuni*, *Salmonella enterica*, enteropathogenic *Escherichia coli* (EPEC), *Proteus mirabilis*, or *Yersinia enterocolitica* also *target E-cadherin during infection* [22,23,24], leading to the hypothesis that bacteria utilize HtrA proteases to promote infectious diseases via a local opening of intercellular adhesions [21].

Based on these previous findings, we hypothesize that HtrA-mediated E-cadherin cleavage is a prevalent mechanism, which is also implicated in the infection process in other mammals. Using *H. pylori* HtrA as a prime example for active HtrA proteases, we investigated the hypothesis that cleavage of E-cadherin from other species represents a gateway for bacterial pathogens in non-human organisms.

## 2. Materials and Methods

### 2.1. Plasmids

To generate the E-cadherin (Cdh1) expression constructs encoding species-specific Cdh1^FL^-GST-His fusion proteins, the sequences for the extracellular domains of *cdh1* genes were amplified from the genomes of indicated animals (HsCdh1 from *Homo sapiens*, Asp155–Ala709, accession no. NP_004351.1 UniProt: P12830); ClCdh1 from *Canis lupus* familiaris, Asp157–Ala712, accession no. NP_001274054.1 UniProt: F1PAA9; MfCdh1 from *Macaca fascicularis*, Asp155–Ala709, accession no. XP_005592416.1 UniProt: A0A2K5V299; MmCdh1 from *Mus musculus*, Asp157–Ala711, accession no. NP_033994.1 UniProt: P09803) lacking the TM and the IC domains (Table 1). NheI/SacII or HindIII/SacII digested PCR-generated inserts were ligated into a pEGFPN_3_-based expression vector and fused to a GST- and His_6_-double tag allowing purification and detection by Western blotting. Full length *cdh1* genes were cloned into the pEGFPN_3_ expression vector to express full length Cdh1 proteins C-terminally fused to GFP in eukaryotic cells (Table 1).

### 2.2. Recombinant Proteins

For the purification of recombinant glycosylated HsCdh1, ClCdh1, MfCdh1, or MmCdh1 containing a C-terminal GST-His_6_-tag, AGS cells were transfected for 16 h with plasmid DNA using polyethylenimine (PEI) in a 1:3 DNA:PEI ratio. The supernatant of transfected cells was collected every 24 h for the following 3 days, centrifuged at 350× *g* at 4 °C for 5 min and pooled in a sterile flask. Recombinant Cdh1-GST-His_6_-tagged proteins were bound to glutathione sepharose beads (GE Healthcare Life Sciences, Vienna, Austria) and eluted with 10 mM reduced glutathione (Carl Roth, Karlsruhe, Germany) for 16 h at 4 °C. Finally, purified proteins were dialyzed in 50 mM HEPES, pH 7.4, 150 mM NaCl and 900 µM CaCl_2_. Recombinant HtrA from *H. pylori* strain Hp26695 (HpHtrA, G18-K475, UniProt G2J5T2, EC 3.4.21.107) and its isogenic inactive mutant (S_221_A) was purified as described previously [25]. Briefly, *E. coli* BL21 were transformed with an HtrA expression vector and grown in terrific broth (TB) medium to an OD_600_ ~0.7 at 37 °C. Expression of GST-HtrA was induced by addition of 100 µM IPTG for 3 h at 30 °C. Bacteria were harvested by centrifugation, resuspended in PBS and lysed by sonication on ice 6 times for 30 s at 50% power (Sonoplus Ultraschall Homogenisator HD270, Bandelin electronic GmbH, Berlin, Germany). GST-tagged HtrA proteins were bound to glutathione sepharose beads (GE Healthcare Life Sciences, Vienna, Austria) and GST-tag was removed by addition of PreScission protease (GE Healthcare Life Sciences, Vienna, Austria) for 16 h at 4 °C. Recombinant HtrA was eluted and dialyzed against 50 mM HEPES (pH 7.4) and 150 mM NaCl. Purity of recombinant proteins is routinely checked with SDS-PAGE and staining with Coomassie Brilliant Blue G250 (Carl Roth, Karlsruhe, Germany).

### 2.3. In Vitro Cleavage Assays

For in vitro cleavage assays, 100 ng recombinant Cdh1 proteins derived from *Homo sapiens*, *Macaca fascicularis*, *Canis lupus familiaris*, and *Mus musculus* were incubated with 20, 50, 100, 250, 500 or 1000 ng active HtrA wt or 1000 ng inactive HtrA S_221_A (SA) in 50 mM HEPES (pH 7.4), 150 mM NaCl and 0.5 mM EGTA for 16 h at 37 °C. To analyze the kinetics, 100 ng recombinant Cdh1 proteins were incubated with 250 ng active HtrA wt or inactive HtrA SA for 1, 2, 4, 8 and 16 h at 37 °C in 50 mM HEPES (pH 7.4), 150 mM NaCl and 0.5 mM EGTA. All in vitro cleavage reactions were performed in a total volume of 20 µL.

### 2.4. SDS-PAGE and Western Blot

Protein samples obtained from in vitro cleavage or infection experiments were separated by SDS-PAGE and blotted on a nitrocellulose membrane. For detection of recombinant Cdh1 proteins, an antibody recognizing the His_6_-tag epitope (Rockland) was used. E-cadherin from whole cell lysates was detected with an antibody recognizing the highly conserved IC domain of E-cadherin (BD Transduction Laboratories™, Heidelberg, Germany). HtrA and CagA were detected using polyclonal sera. GAPDH (Cell Signaling Technology, Frankfurt, Germany) was used as a loading control for whole cell lysates.

### 2.5. Mutagenesis of MKN-28 Cells

For disruption of endogenous Cdh1 expression in MKN-28 cells, a specific oligonucleotide targeting exon ENSE00000844392 of hCdh1 was cloned into the guide RNA plasmid pX330-U6-Chimeric_BB-CBh-hSpCas9 [26]. For design of the single-guide RNA, an online tool made available by the Zhang laboratory of the Massachusetts Institute of Technology was used (Available online: http://CRISPR.mit.edu, accessed on 29 April 2014). The following oligonucleotides were synthesized, annealed, and inserted into the vector after BbsI digestion: gRNA_EC_n1_1/2: 5′- CAC CGC GCC GAG AGC TAC ACG TTC A and gRNA_EC_n1_2/2: 5′- AAA CTG AAC GTG TAG CTC TCG GCG C. Oligonucleotide synthesis and sequencing for confirmation of correct construct sequence was provided by Eurofins. MKN-28 cells were co-transfected with the plasmid pX330 carrying hSpCas9 and the guide RNA. Transfection was performed using PEI, with subsequent selection by incubation with medium containing 30 µg/mL blasticidin. Viable cells were consequently isolated, expanded and verified for Cdh1 expression by sequencing and Western blotting. For stable expression of orthologous Cdh1-GFP MKN-28 *Δcdh1* cells were seeded in 6-well plates at low confluency and incubated for 24 h at 37 °C and 5% CO_2_. Cells were transfected with linearized plasmids using lipofectamine 3000 (Thermo Fisher Scientific, Vienna, Austria) for 48 h according to the manufacturers protocol. Selection of transfected cells was performed with G418 disulfate (Sigma Aldrich, Vienna, Austria).

### 2.6. Cell Culture and Infection Experiments

The gastric epithelial cell lines MKN-28 expressing Cdh1-GFP orthologues were grown in RPMI-1640 (Sigma Aldrich, Vienna, Austria) supplemented with 10% FCS (Biowest, Vienna, Austria), 1% L-glutamine (Biowest, Vienna, Austria), 10 mM HEPES (Carl Roth, Karlsruhe, Germany), and 400 µg/mL G418 disulfate in a humidified atmosphere at 37 °C and 5% CO_2_. For infection experiments, cells were seeded in 6-well plates three days prior to infection. *H. pylori* strains P12 wild type (wt), N6 wt, N6 *ΔhtrA* and, N6 *ΔhtrA/htrA wt* [27,28] were cultured on agar plates containing 10% horse serum (Biowest, Vienna, Austria) under microaerophilic conditions at 37 °C for 16 h before infection. Cells were washed extensively with PBS to remove G418 disulfate solution from the cells and cells were starved 60 min before infection. MKN-28 cells were infected with *H. pylori* P12 wt, N6 wt, N6 *ΔhtrA*, N6 *ΔhtrA/htrA wt* at a MOI (multiplicity of infection) of 100 for 24 h or left untreated (mock). For preparation of whole cell lysates, MKN-28 cells were washed two times with PBS and harvested in lysis buffer (20 mM Tris pH 7.5, 1 mM EDTA, 100 mM NaCl, 1% Triton X-100, 0.5% DOC, 0.1% SDS, 0.5% NP-40) supplemented with 1× PIT (protease inhibitor cocktail complete EDTA-free tablets, Roche), 1 mM sodium molybdate, 20 mM sodium fluoride, 20 mM β-glycerophosphate, 1 mM sodium orthovanadate. Whole cell lysates were cleared from cell debris by centrifugation at 16,000× *g* for 10 min at 4 °C.

### 2.7. Immunofluorescence and STED Microscopy

For immunofluorescence, cells were seeded on poly-L-lysine (Sigma Aldrich, Vienna, Austria) coated cover slips (Carl Roth, Karlsruhe, Germany) and infected with *H. pylori* P12 wt, N6 wt, N6 *ΔhtrA*, at a MOI of 50 for 24 h or left untreated (mock). Cells were washed twice with PBS containing CaCl_2_ and MgCl_2_ and fixed with ice-cold methanol at −20 °C for 10 min. To localize Cdh1, cells were stained with an antibody targeting the IC domain of E-cadherin (BD Transduction Laboratories™, Heidelberg, Germany), which is highly conserved in all Cdh1 variants. *H. pylori* was detected using a polyclonal serum raised against *H. pylori* lysate (gift from Rainer Haas, Munich) and nuclei were stained with 4′,6-diamidino-2-phenylindole (DAPI) (Carl Roth, Karlsruhe, Germany). After incubation with secondary antibodies coupled to STAR-RED or STAR580 dyes (Abberior, Göttingen, Germany) cover slips were mounted on glass microscopy slides using antifade mounting medium (Abberior, Göttingen, Germany) and fluorescence was detected using a Zeiss Observer Z1 fluorescence microscope equipped with an Abberior Instruments STEDYCON unit for confocal and super-resolution STED microscopy.

### 2.8. Colony-Forming Unit Assay

MKN-28 cells were infected with *H. pylori* P12 wt, N6 wt and N6 *ΔhtrA* and N6 *ΔhtrA*/*htrA* wt at MOI 50 for 6 h. To remove unbound bacteria, cells were washed 3 times with pre-warmed PBS and incubated in lysis solution (PBS containing 0.05% saponin) for 15 min at 37 °C and 5% CO_2_. The resulting suspension was diluted in 10-fold serial steps and three appropriate dilutions were plated on GC agar plates in duplicates. After 3–5 days of incubation under microaerophilic conditions, the number of cell-bound bacteria was quantified as colony forming units (CFU). The cell binding assay was repeated four times.

### 2.9. Statistical Analysis

Statistical analysis of the adherence assay was performed using ordinary one-way ANOVA and the Tukey’s post hoc test with the GraphPad Prism software (version 8.0.2). Four independent experiments were analyzed. *p* values > 0.05 were not considered as statistically significant. Densitometric quantification of cadherin full-length bands was performed with the ImageLab Software (version 3.0.1) and are expressed as mean percentage ± SD, with E-cadherin treated with inactive HtrA SA set to 100%. Statistical analysis was performed on raw data using the student’s *t*-test (paired two tailed). Three independent experiments were analyzed. *p* values > 0.05 were not considered statistically significant.

## 3. Results and Discussion

### 3.1. HtrA Cleaves Different Cadherin Orthologues

**Figure 1:**
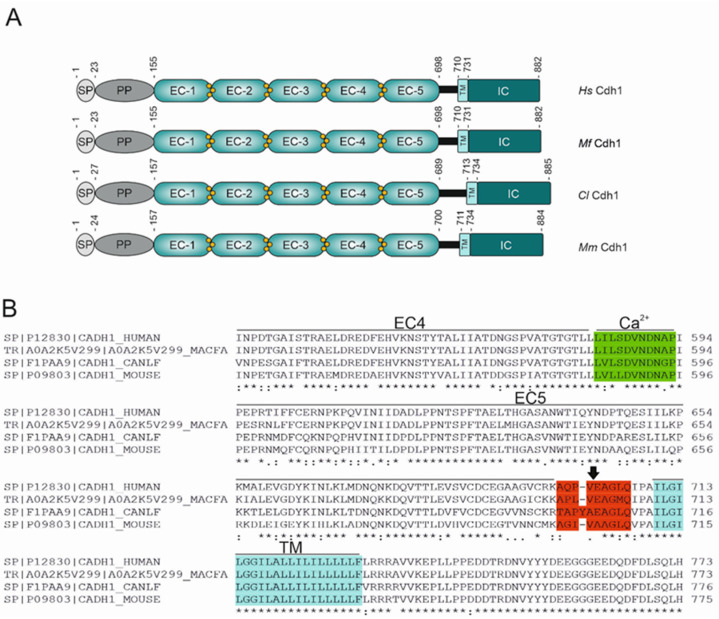
Cdh1 orthologues share high sequence homologies in HtrA cleavage sites. (**A**) Models of the domain structure of the Cdh1 expressed by *Homo sapiens* (*Hs*), *Macaca fascicularis* (*Mf*), *Canis lupus familiaris* (*Cl*) and *Mus musculus* (*Mm*). Calcium binding regions are located in between the EC domains with calcium ions shown as yellow spheres. SP, signal peptide; PP, pro-peptide; EC, extracellular domain; TM, transmembrane domain; IC, intracellular domain. (**B**) Alignment of the amino acid sequence of Cdh1 orthologues spanning the region between the EC4, HtrA signature sites (highlighted in green), EC5, linker region (highlighted in red), identified HtrA cleavage site (black arrow), and the TM domain (highlighted in blue).

To investigate whether E-cadherin from other mammal species serves as an HtrA substrate, we selected E-cadherin (Cdh1) expressed by *Macaca fascicularis (Mf)*, *Canis lupus familiaris* (*Cl*), or *Mus musculus* (*Mm*) and compared them with Cdh1 from *Homo sapiens* (*Hs*) (Table 2). All Cdh1 orthologues share an N-terminal signal peptide (SP) and a downstream pro-peptide (PP) that are removed post-translationally during transport to the cell surface. The characteristic feature of all Cdh1 orthologues is the extracellular domain (EC), which consists of tandem repeats (EC1-EC5) with interspaced negatively charged amino acid sequence motifs DRE, DXNDNXP and DXD. These motifs bind calcium ions that are required for the adhesive properties of cadherin proteins [29,30]. The extracellular domain is followed by a short linker region, a transmembrane (TM), and an intracellular (IC) domain (Figure 1A,B). The EC domain of human Cdh1 shares 78–95% sequence identity and 85–97% similarity (Table 2). Comparing the amino acid sequences of Cdh1 orthologues, a high degree of conservation in the calcium binding sites was revealed (Figure 1B, highlighted in green), while more sequence variability was found in the linker regions (Figure 1B, highlighted in red). The conserved [VITA]-[VITA]-x-x-D-[DN] sequence pattern in calcium binding sites was initially identified as important signature motifs for *H. pylori* HtrA cleavage leading to a unique fragmentation pattern of Cdh1 [31]. However, in tissues with intact intercellular adhesions, these sites are masked by bound calcium ions, which efficiently prevents the fragmentation of the extracellular domain of Cdh1 [31,32]. The amino acid sequence AQPV^↓^EAG within the linker region (Figure 1B, highlighted in red) was recently determined as a potent HtrA cleavage site targeted by *H. pylori* during infection. Cleavage after the hydrophobic valine in position P1 was identified to be responsible for the formation of the soluble 90 kDa fragment observed after *H. pylori* infection of polarized gastric epithelial cells [33], suggesting that this site is the major site in the extracellular E-cadherin domain for HtrA proteases. However, this cleavage site in the linker region of E-cadherin exhibits sequence variations in Cdh1 orthologues from humans, monkeys, mice, and dogs (Figure 1B), implying that HtrA targets E-cadherin orthologues with altered efficiency in these species.

**Figure 2:**
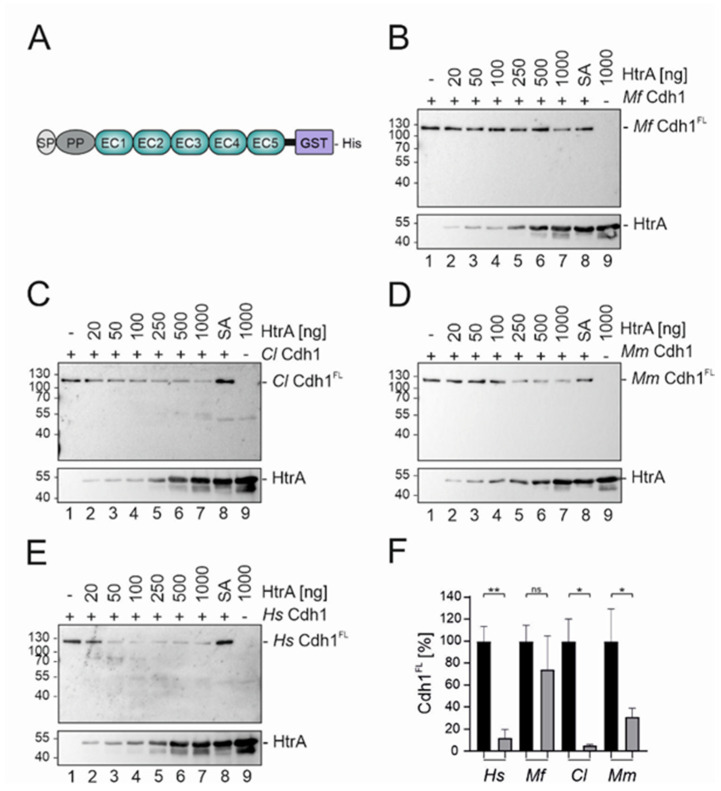
Cdh1 variants are efficiently cleaved by HtrA. (**A**) Scheme of the recombinant Cdh1 proteins. 100 ng of recombinant *Mf* Cdh1 (**B**)*, Cl* Cdh1 (**C**), *Mm* Cdh1 (**D**), and *Hs* Cdh1 (**E**) were incubated with the indicated amounts of recombinant active HtrA for 16 h at 37 °C (lanes 2–7). As a control, recombinant Cdh1 were incubated with 1000 ng of inactive HtrA (SA, lane 8) or left untreated (lane 1). To control possible antibody cross-reactions, HtrA alone was loaded (lane 9). The different Cdh1 full-length (FL) versions were detected using an antibody recognizing the C-terminal His_6_-tag. HtrA was detected by using a polyclonal HtrA antibody. (**F**) The amount of Cdh1^FL^ treated with 1000 ng recombinant active HtrA (grey) or inactive HtrA (black) was quantified by blot densitometry. Data are presented as percent means of Cad^FL^ ± SD (*n* = 3), with the cadherin treated with the inactive HtrA set at 100% and compared with the signal intensity of Cdh1^FL^ treated with 1000 ng of active HtrA. Asterisks indicate statistically significant differences (** *p* < 0.01; * *p* < 0.05; ns, not significant).

For the experimental analyses of HtrA-mediated cleavage of these Cdh1 orthologues, recombinant proteins with the characteristic glycosylation patterns of the native proteins were required. Sequences encoding the extracellular Cdh1 domains, including SP and PP, but lacking the TM and IC domains were cloned into eukaryotic expression systems to generate secreted and glycosylated Cdh1 orthologues fused to a C-terminal GST-His_6_-tag (Figure 2A). Human gastric epithelial AGS cells lacking endogenous Cdh1 expression [34] were transiently transfected and Cdh1-GST-His_6_ proteins were purified from the supernatants. Next, 100 ng of purified recombinant *Mf* (Figure 2B), *Cl* (Figure 2C), or *Mm* Cdh1 (Figure 2D) was incubated for 16 h with increasing amounts of recombinant *H. pylori* HtrA wild type (wt), which serves as a prime examples for active HtrA protease. *Hs* Cdh1 was also included in this study, serving as a well-established reference (Figure 2E). As negative controls, Cdh1 proteins were incubated without HtrA (-) or with 1000 ng proteolytic inactive HtrA S_221_A (SA) (Figure 2B–E, lanes 1 and 8). Also, 1000 ng HtrA wt served as control for antibody cross reaction (Figure 2B–E, lane 9). Compared to *Hs* Cdh1 (Figure 2E), high concentrations of HtrA were necessary to induce *Mf* Cdh1 cleavage (Figure 2B). The strongest effect was observed after incubation of *Mf* Cdh1 with 1000 ng of HtrA as reflected by the decrease of full-length *Mf* Cdh1 (*Mf* Cdh1^FL^) (Figure 2B, compare lanes 2–7 with lanes 1 and 8). In contrast, low concentrations of HtrA were sufficient to cleave *Cl* and *Mm* Cdh1^FL^, which was further enhanced using increasing amounts of HtrA (Figure 2C,D). The signals from Western blot analyses were quantitated from three independent experiments and confirmed the efficient HtrA cleavage activity using *Hs*, *Cl*, and *Mm* Cdh1 as substrates, and the lower activity towards *Mf* Cdh1 (Figure 2F). This result is unexpected, as *Mf* Cdh1 and *Hs* Cdh1 show the strongest homology. Nevertheless, individual changes of amino acids in the EC domain may influence the accessibility of Cdh1 for HtrA, affecting the in vitro cleavage activity.

**Figure 3:**
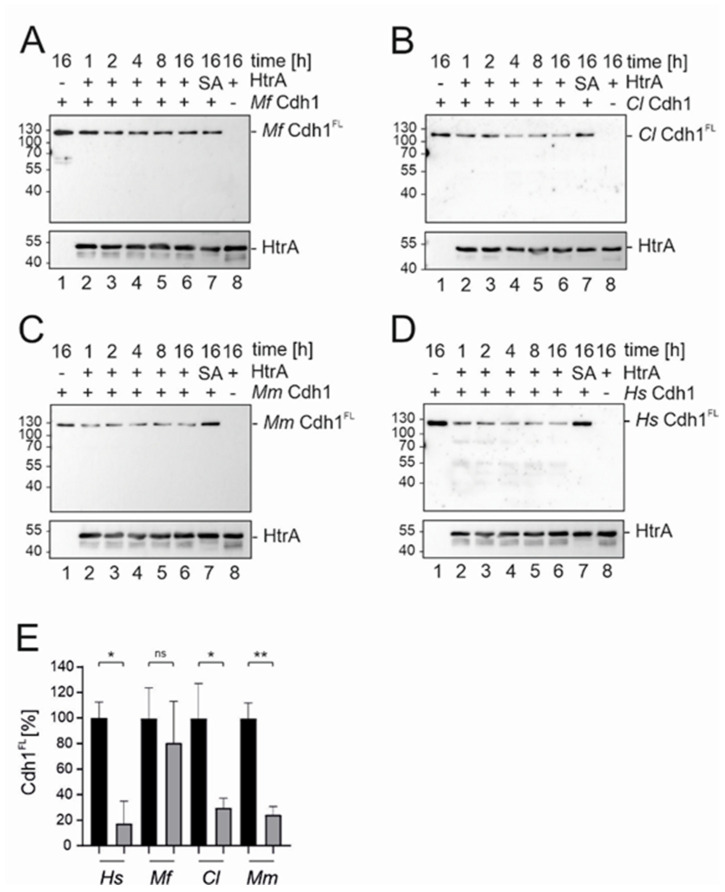
Kinetics of HtrA-mediated Cdh1 cleavage. 100 ng of recombinant *Mf* Cdh1 (**A**), *Cl* Cdh1 (**B**), *Mm* Cdh1 (**C**), and *Hs* Cdh1 (**D**) were incubated with 250 ng recombinant active HtrA for the indicated time points at 37 °C (lanes 2–6). As a control, recombinant Cdh1 was incubated for 16 h with inactive HtrA (SA, lane 7) or left untreated (lane 1). To control antibody cross-reactions, HtrA alone was loaded (lane 8). The different Cdh1^FL^ variants were detected using an antibody recognizing the C-terminal His_6_-tag. HtrA was detected using a polyclonal HtrA antibody. (**E**) The amount of Cdh1^FL^ treated for 16 h with recombinant active HtrA (grey) or inactive HtrA SA (black) was quantified by blot densitometry. Data are presented as percent means of Cad^FL^ ± SD (*n* = 3), with the cadherin treated with the inactive HtrA SA set at 100% and compared with the signal intensity of Cdh1^FL^ treated with active HtrA for 16 h. Asterisks indicate statistically significant differences (** *p* < 0.01; * *p* < 0.05; ns, not significant).

These findings were further investigated through the analysis of the kinetics of proteolytic activities by additional in vitro cleavage experiments. Recombinant *Mf*, *Cl*, and *Mm* Cdh1 were incubated with 250 ng HtrA for the indicated periods of time (Figure 3A–C) and compared to the kinetics of HtrA-mediated *Hs* Cdh1 cleavage (Figure 3D). Again, the amount of *Mf* Cdh1^FL^ only weakly decreased after incubation with HtrA (Figure 3A). Even after 16 h there was no significant difference between HtrA wt and HtrA SA treated *Mf* Cdh1 (Figure 3A,E). Considering *Cl* Cdh1^FL^ (Figure 3B) and *Mm* Cdh1^FL^ (Figure 3C), a strong decrease could be observed already after one hour of incubation with HtrA (Figure 3B,C, lanes 6 and 7). Quantification demonstrated statistically significant HtrA wt-mediated *Cl* Cdh1 and *Mm* Cdh1 cleavage compared to the treatment with the inactive HtrA SA after 16 h (Figure 3E). The kinetics and the extent of HtrA-mediated cleavage of *Cl* Cdh1 and *Mm* Cdh1 were comparable to HtrA-mediated cleavage of *Hs* Cdh1, which was included as a control (Figure 3D,E).

### 3.2. Cleavage of Orthologous E-Cadherin after Infection of Epithelial Cells

**Figure 4:**
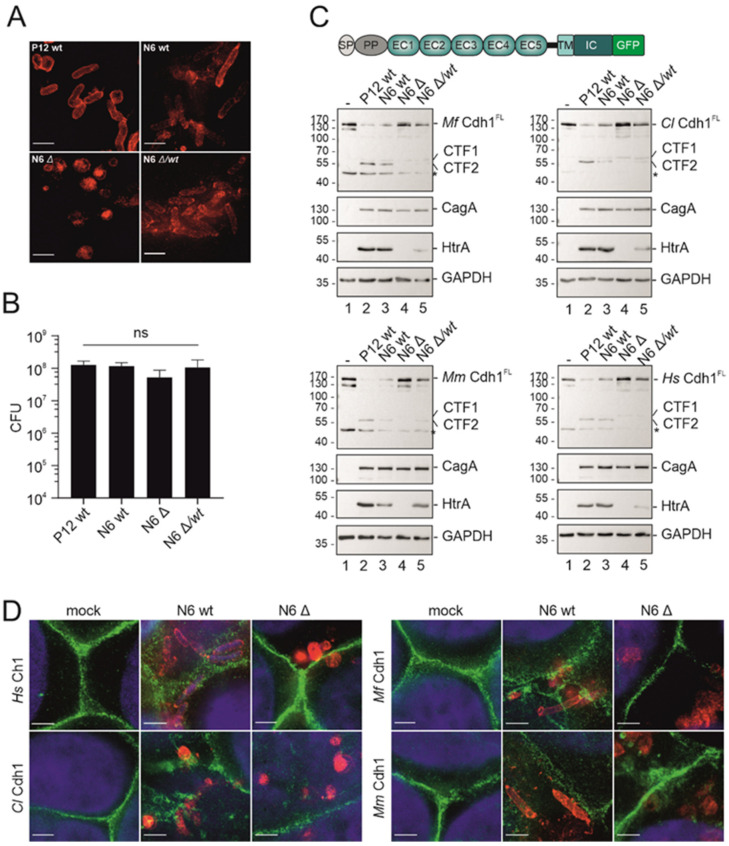
Infection of gastric epithelial cells expressing Cdh1 orthologues. (**A**) Representative STED images of *H. pylori* P12 wt, N6 wt, N6 *ΔhtrA,* and N6 *ΔhtrA/htrA wt* bacteria attached to gastric epithelial cells stained using a polyclonal serum raised against *H. pylori* lysate. Scale bar, 2 µm. (**B**) MKN-28 wt cells were infected with *H. pylori* P12 wt, N6 wt, N6 *ΔhtrA,* and N6 *ΔhtrA/htrA wt* for 6 h and the number of bacterial cells adhering to MKN-28 cells was quantified and are presented as mean values ± SD (*n* = 4). CFU, colony forming unit; ns, not significant. (**C**) MKN-28 cells stably expressing Cdh1 proteins as indicated were infected with *H. pylori* P12 wt, N6 wt, N6 *ΔhtrA,* and N6 *ΔhtrA/htrA wt* for 24 h. Non-infected cells served as control. Whole cell lysates of infected cells were analyzed by Western blotting for Cdh1 full length (Cdh1^FL^) and the C-terminal fragments CFT1 and CTF2 using an antibody recognizing the intracellular domain of Cdh1 (first panel). CagA and HtrA were detected as loading controls for *H. pylori* (second and third panel). Loading control for MKN-28 cells was performed by detection of GAPDH (fourth panel). *, unspecific band. (**D**) Representative STED images of MKN-28 cells stably expressing Cdh1 proteins (green) derived from *Hs*, *Mf*, *Cl*, and *Mm* infected with *H. pylori* N6 wt and N6 *ΔhtrA* bacteria (red), or uninfected controls (mock); Nuclei were stained using DAPI (blue). Scale bars, 2 µm.

In vitro cleavage experiments certainly do not reflect the physiological situation during infection, which is strongly influenced by the dynamic and complex structure of the adhesion complexes. Hence, we investigated the cleavage of Cdh1 orthologues in a bacterial infection model using the *H. pylori* wt strain P12 and N6, an isogenic N6 *htrA* deletion mutant (N6 ∆) and an *htrA*-complemented mutant (N6 ∆/wt) [27]. Genomic deletion of *htrA* genes in pathogens has frequently been associated with a less pathogenic phenotype [35,36]. Hence, MKN-28 cells were infected with *H. pylori* strains followed by immunofluorescence staining using an anti-*H. pylori* serum. Super-resolution imaging using a STED microscope revealed a rod-shaped morphology for P12 wt, N6 wt, and N6 ∆/wt as expected, but a coccoid phenotype of N6 ∆ (Figure 4A). This observation suggests that HtrA might play a determinant role in bacterial morphology either through absence of the periplasmic chaperone function of HtrA or through direct processing of bacterial factors, which are important for the cellular phenotype. An effect of HtrA proteases on bacterial cell shape was described for *Mycobacterium smegmatis* [37] and the cyanobacterium *Synechocystis* sp. PCC 6803, where the deletion of the *htrA* gene caused a defect in the biosynthesis of the outer cell layers [38]. Hence, further investigations are necessary to examine the impact of HtrA on the regulation of the cell wall.

HtrA expression has repeatedly been associated with bacterial adhesions to host cells [35,36]. We observed only a slight effect of H. pylori HtrA expression on the adherence to MKN-28 cells (Figure 4B) indicating that the htrA knockout mutant can equally well colonize epithelial cells as previously demonstrated [28]. To analyze the impact of HtrA-positive H. pylori on cleavage of Cdh1 orthologues, we established a novel infection model using the partially polarized gastric epithelial cell line MKN-28. The endogenous Cdh1 expression was interrupted by CRISPR/Cas9-mediated genomic deletion of the hcdh1 gene and orthologous Cdh1 proteins were stably re-expressed with full-length Hs, Mf, Cl, or Mm CDH1 genes fused to a C-terminal GFP tag (Figure 4C). We then investigated the Cdh1 fragments of variants in whole cell lysates of MKN-28 cells after colonization with the H. pylori strains P12 wt, N6 wt, N6 ∆, as well as N6 ∆/wt [27]. We observed a strong cleavage of Mf Cdh1 after infection with HtrA-positive H. pylori strains, which resulted in the reduction of full-length Mf Cdh1 and the appearance of the C-terminal fragments CFT1 and CTF2 [17]. The detected fragmentation pattern of Cdh1 is characteristic for H. pylori infections. Full-length Cdh1FL can be cleaved in a soluble extracellular domain N-terminal fragment, which is dependent on HtrA and largely independent of host proteases [17,21]. Removal of the extracellular E-cadherin domain generates a membrane-anchored 40 kDa C-terminal fragment CTF1, which can be further processed by intracellular host proteases into a soluble 33 kDa CTF2 fragment [39]. Calpain was identified to induce a specific, inactivating proteolytic cleavage within the cytoplasmic domain of Cdh1 in prostate and mammary epithelial cells [40]. Since H. pylori can activate calpain [41], we assume that activated calpain mediates the intracellular cleavage of Cdh1.

Importantly, infection with N6 *∆htrA* mutant did not fragment *Mf* Cdh1, while the complemented N6 ∆/wt enhanced *Mf* Cdh1 cleavage (Figure 4C, upper left panel), which is in a slight contrast to the in vitro cleavage experiments. The discrepancy between these data cannot be simply explained by the HtrA cleavage sites in the calcium binding site or the linker regions, which are highly homologous to *Hs* Cdh1 (Figure 1B). It has been well established that the binding of calcium to Cdh1 and formation of homophilic interactions of the ectodomains in *cis* and *trans* elongate the curved Cdh1 structure, forming a three-dimensional extracellular net of interacting ectodomains [2,42]. Therefore, we propose that the conformation and structure of Cdh1 proteins are important aspects in the recognition of Cdh1 by HtrA on the cell surface. Similar results were observed for the infection of MKNK-28 cells expressing either *Cl* Cdh1 (Figure 4C, upper right panel), *Mm* Cdh1 (Figure 4C, lower left panel), or *Hs* Cdh1 (Figure 4C, lower right panel), which was included as a control. Detection of HtrA was performed to verify HtrA expression in *H. pylori* strain. CagA was detected as a control for equal bacterial adherence and GAPDH served as a loading control (Figure 4C).

The consequences of Cdh1 cleavage were verified by super-resolution microscopy to monitor the changed localization of Cdh1 proteins upon infection with HtrA-positive bacteria. Intact Cdh1-based adherens junctions are membrane-located and the loss of the ectodomain of Cdh1 leads to a disintegration of the Cdh1 complex followed by the internalization of the intracellular Cdh1 domain in vesicles [43]. Stably expressing Cdh1-GFP MKN-28 cells were infected with *H. pylori* wt or the isogenic *htrA* deletion mutant and the cellular distribution of *Hs* Cdh1, *Mf* Cdh1, *Cl* Cdh1, and *Mm* Cdh1 was analyzed (Figure 4D). In non-infected cells, Cdh1 proteins were clearly located in the cell membrane. After infection with *H. pylori* wt, membrane localization of Cdh1 proteins was lost or became highly diffuse at sites where bacteria attached to cells. These data imply that HtrA-mediated cleavage is a local process and can mainly be observed in the direct vicinity of the bacteria. Cdh1 proteins appear in speckles indicating internalization of truncated Cdh1 CTFs in vesicles [43]. If truncated CTF fragments are internalized after HtrA-mediated shedding from the cell surface or after additional cleavage of the intracellular domain via calpain is not known, but will be investigated in the future.

## 4. Conclusions

In this study it was shown that HtrA targets Cdh1 orthologues from *Macaca fascicularis*, *Canis lupus familiaris*, and *Mus muculus* to a similar extent as human Cdh1. *H. pylori* is a human pathogen that exclusively colonizes human stomachs, but a large number of non-*H. pylori* species were detected in animals, which were not extensively studied yet. In conclusion, HtrA proteins are highly conserved in the *Helicobacter* genus. Therefore, we propose that HtrA-mediated Cdh1 shedding is a relevant process in the pathogenesis induced by other *Helicobacter* species in non-human organisms.

## Figures and Tables

**Table 1:** Constructs used in the study.

Constructs	aa		Primer
HsCdh1^EC^-GST-His	1–709	forward	5′–GATC**GCTAGC**CACCATGGGCCCTTGGAG–3′
		reverse	5′–GATC**CCGCGG**TGGCAGGAATTTGCAATC–3′
ClCdh1^EC^-GST-His	1–712	forward	5′–GATC**GCTAGC**CACCATGGGCCCTCGGTAC–3′
		reverse	5′–GATC**CCGCGG**TGGCAGGAACCTGCAAG–3′
MfCdh1^EC^-GST-His	1–709	forward	5′–GATC**GCTAGC**CACCATGGGCCCTTGGAG–3′
		reverse	5′–GATC**CCGCGG**TGGCAGGAACCTGCAAG–3′
MmCdh1^EC^-GST-His	1–711	forward	5′–GATC**AAGCTT**CACCATGGGAGCCCGGTG–3′
		reverse	5′–GATC**CCGCGG**TGGCAGGAACTTGCAATC–3′
HsCdh1^FL^-GFP	1–882	forward	5′–GATC**CCGCGG**CTAATGATGATGATGATGATGATCCGATTTTGGAGGATG–3′
		reverse	5′–CTAG**CCGCGG**TGTCGTCCTCGCCG–3′
ClCdh1^FL^-GFP	1–885	forward	5′–GATC**GCTAGC**CACCATGGGCCCTCGGTAC–3′
		reverse	5′–CTAG**CCGCGG**TGTCGTCCTCGCCACC–3′
MfCdh1^FL^-GFP	1–882	forward	5′–GATC**GCTAGC**CACCATGGGCCCTTGGAG–3′
		reverse	5′–CTAG**CCGCGG**TGTCATCCTCGCCGC–3′
MmCdh1^FL^-GFP	1–884	forward	5′–GATC**AAGCTT**CACCATGGGAGCCCGGTG–3′
		reverse	5′–CTAG**CCGCGG**TGTCGTCCTCACCACCG–3′

**Table 2:** Comparison of the extracellular domain of Cdh1 orthologues.

Name	UniProt No.	Gene	Species	% Identity ^1^	% Similarity
E-cadherin	P12830	*CDH1*	*Homo sapiens*	100	100
E-cadherin	A0A2K5V299	*CDH1*	*Macaca fascicularis*	95	97
E-cadherin	F1PAA9	*CDH1*	*Canis lupus familiaris*	79	87
E-cadherin	P09803	*CDH1*	*Mus musculus*	78	85

^1^ Sequence alignment was performed for the annotated extracellular domain of Cdh1 proteins including the signal peptide and pro-peptide.

## Data Availability

Data available on request from the authors.
